# Multiple Mechanistic Action of Brevinin-1FL Peptide against Oxidative Stress Effects in an Acute Inflammatory Model of Carrageenan-Induced Damage

**DOI:** 10.1155/2022/2615178

**Published:** 2022-09-05

**Authors:** Jinwei Chai, Junfang Liu, Maolin Tian, Hang Liao, Jiena Wu, Jianpeng Xie, Shian Lai, Guoxiang Mo, Xin Chen, Xueqing Xu

**Affiliations:** ^1^Department of Pulmonary and Critical Care Medicine, Zhujiang Hospital, Southern Medical University, Guangzhou, China; ^2^Guangdong Provincial Key Laboratory of New Drug Screening, School of Pharmaceutical Sciences, Southern Medical University, Guangzhou, China; ^3^Department of Molecular Chemistry and Biochemistry, Faculty of Science and Engineering, Doshisha University, Kyotanabe, Japan; ^4^College of Life Sciences, Nanjing Agricultural University, Nanjing, China

## Abstract

Amphibian skin is acknowledged to contain an antioxidant system composed of various gene-encoded antioxidant peptides, which exert significant effects on host defense. Nevertheless, recognition of such peptides is in its infancy so far. Here, we reported the antioxidant properties and underlying mechanism of a new antioxidant peptide, brevinin-1FL, identified from *Fejervarya limnocharis* frog skin. The cDNA sequence encoding brevinin-1FL was successfully cloned from the total cDNA of *F. limnocharis* and showed to contain 222 bp. The deduced mature peptide sequence of brevinin-1FL was FWERCSRWLLN. Functional analysis revealed that brevinin-1FL could concentration-dependently scavenge ABTS^+^, DPPH, NO, and hydroxyl radicals and alleviate iron oxidation. Besides, brevinin-1FL was found to show neuroprotective activity by reducing contents of MDA and ROS plus mitochondrial membrane potential, increasing endogenous antioxidant enzyme activity, and suppressing H_2_O_2_-induced death, apoptosis, and cycle arrest in PC12 cells which were associated with its regulation of AKT/MAPK/NF-*κ*B signal pathways. Moreover, brevinin-1FL relieved paw edema, decreased the levels of TNF-*α*, IL-1*β*, IL-6, MPO, and malondialdehyde (MDA), and restored catalase (CAT) and superoxide dismutase (SOD) activity plus glutathione (GSH) contents in the mouse injected by carrageenan. Together, these findings indicate that brevinin-1FL as an antioxidant has potent therapeutic potential for the diseases induced by oxidative damage. Meanwhile, this study will help us further comprehend the biological functions of amphibian skin and the mechanism by which antioxidants protect cells from oxidative stress.

## 1. Introduction

Free radicals including superoxide anion radical, peroxyl radical, and hydroxyl radical are unstable and indispensable intermediates of aerobic metabolism during respiration in organisms. In general, they are highly reactive with the other groups or substances in the body due to their single and unbalanced electrons. Consequently, free radicals can trigger a cascade of damaging reactions such as lipid peroxidation as well as protein and DNA oxidation, affecting cellular signaling and culminating in cell damage and death [1, 2]. ROS are effectually obliterated by the antioxidant defense system including nonenzymatic factors and antioxidant enzymes under normal conditions. But, under pathological conditions, the homeostasis between the generation and scavenging of ROS is broken *in vivo*, which is generally acknowledged to be involved in some health disorders such as diabetes mellitus, cancer, atherosclerosis, aging, and neurodegenerative and inflammatory diseases [2, 3]. Thus, effectively scavenging excessive free radicals or preventing their generation has been momentous methods for the prevention and treatment of such diseases. Many synthetic antioxidants, such as vitamin C, butylated hydroxyanisole, and propyl gallate, are used for retarding lipid peroxidation. Yet their potential health hazards and low stability limit their further medical application [4]. Hence, the isolation and identification of natural antioxidants which can scavenge free radicals and protect cells from oxidative damage have been of great interest among researchers [5].

Amphibian has evolved an efficient antioxidant defense system in their skin to antagonize nonbiological injuries from ROS in their environments. Antioxidant peptides are important components of the amphibian skin antioxidant system, and more than 40 peptides with free radial scavenging functions have been identified by purification, proteomic analysis, or cDNA trapping from *R. catesbeiana*, *O. livida*, *R. pleuraden*, *N. parkeri*, *O. macrotympana*, *O. margaretae*, *O. andersonii*, *O. livida*, *O. schmackeri*, *O. wuchuanensis*, *and O. hejiangensis* [6, 7]. *Fejervarya limnocharis* is a medium-sized frog (30-60 mm) inhabiting throughout East, Southeast, and South Asia [8, 9]. Except that a lectin-like peptide inhibiting HIV-1 entry has been identified by us, there is no any bioactive peptide reported from this species [10]. In this work, we firstly characterized brevinin-1FL from the skin of frog *F. limnocharis* which showed potent antioxidant activity *in vitro*. Then, we explored the protective effects of brevinin-1FL against H_2_O_2_-induced ROS generation, oxidative stress, and cytotoxic effect in PC12 cells for evaluating its pharmaceutical potential. Finally, we conducted animal experiment to test its antioxidant and anti-inflammatory activities *in vivo*. To the best of our knowledge, it is the first report about an antioxidant peptide from *F. limnocharis.*

## 2. Materials and Methods

### 2.1. Animals and Ethical Statement

Male and female adult *F. limnocharis* frogs (*n* = 3) obtained from the countryside of Guangzhou, Guangdong Province, China (23.12°N, 113.28°E) are not a protected species without the need for specific permissions. After collection, the frogs were humanely euthanized using CO_2_, and the skin was subsequently sheared and stored in liquid nitrogen until use. Kunming mice (4 weeks, 18-20 g) were bought from the Laboratory Animal Center of Southern Medical University (Guangdong, China) and maintained in plastic cages under standard conditions at 25 ± 2°C and 55 ± 10% humidity, with free access to food and water on a 12 h light/dark rhythm. The Animal Care and Use Ethics Committee of Southern Medical University (no. L2018254) authorized all protocols and procedures involving live animals which were implemented in light of the international regulations for animal research.

### 2.2. Molecular Cloning and Characterization of cDNA Encoding Brevinin-1FL

The skin mRNA and double-strand cDNA were prepared as previously reported by us [10]. The chemical parameters of brevinin-1FL were analyzed with the ExPASy Bioinformatics Resource Portal (http://www.expasy.org/tools/). The assembled sequences were aligned with ClustalW (http://embnet.vital-it.ch/software/ClustalW.html) on the basis of similarity with previously reported antimicrobial peptides (AMPs) from different amphibian species including brevinin-1FL.

### 2.3. Peptide Synthesis

Brevinin-1FL was synthesized by GL Biochem Ltd. (Shanghai, China) and purified with a C18 reverse-phase HPLC column (SHIMAZU, Ōsumi, Japan) to over 95%, before being lyophilized and further confirmed by MALDI-TOF mass spectrometry (Figure S1). FITC-labeled brevinin-1FL was obtained with a FITC conjugation kit according to the producer's protocol (Sangon, Shanghai, China).

### 2.4. Peptide Internalization Analysis

To ensure whether brevinin-1FL can enter into the cells to exert antioxidant effects, PC12 cells (1 × 10^5^ cells/well) were grown in a 24-well plate overnight and then incubated with FITC-labeled brevinin-1FL at the final concentrations of 2, 4, and 8 *μ*M at 37°C for 6 h before the cells were collected and detected by flow cytometry (BD FACSCanto II, MA, USA). To identify the effects of heparin on peptide internalization, 8 *μ*M brevinin-1FL was preincubated with 10, 20, or 40 *μ*g/mL heparin for 30 min, and then, the mixture was incubated with PC12 cells for 6 h before flow cytometry analysis. To identify the effects of the cellular energy state on the internalization, PC12 cells were preincubated with 10, 20, or 40 *μ*M of sodium azide (NaN_3_) or 12.5, 25, or 50 mM of ammonium chloride (NH_4_Cl) for 30 min and then incubated with 8 *μ*M FITC-labeled brevinin-1FL for another 6 h before flow cytometry analysis. To examine the effects of time and temperature on the internalization, PC12 cells were incubated with 8 *μ*M FITC-labeled brevinin-1FL for 1 h or 6 h at 4°C or 37°C. To identify the effects of H_2_O_2_ on internalization, PC12 cells were incubated with 8 *μ*M FITC-labeled brevinin-1FL in the presence of 0.25 mM H_2_O_2_ for 6 h before flow cytometry analysis. All experiments were performed in triplicate.

### 2.5. Antioxidant Activity Measurement in Vitro

The ABTS radical scavenging activity was measured with a commercial kit according to the manufacturer's instruction (Beyotime, Shanghai, China). Briefly, 10 *μ*L brevinin-1FL (0-20 *μ*M) and 200 *μ*L ABTS working solution were mixed in a 96-well plate and placed at room temperature for 21 min. 10 *μ*L of distilled water was used as the negative control. The absorbance was measured at 734 nm with a microplate reader (Infinite M1000 Pro, Tecan Company, Switzerland). ABTS radical scavenging activity was computed as follows: ABTS scavenging activity (%) = (*A*_blank_ − *A*_sample_)/*A*_blank_ × 100, where *A*_blank_ is the absorbance of ABTS solution with distilled water and *A*_sample_ is the absorbance in the presence of brevinin-1FL. All samples were analyzed in triplicate and averaged.

The 2,2-diphenyl-1-picrylhydrazyl (DPPH) radical-scavenging activity was measured with the method described previously by us [11]. A 10 *μ*L aliquot of brevinin-1FL (0-20 *μ*M) was mixed with a 100 *μ*L methanolic solution of DPPH radical at the final concentration of 0.2 mM before being shaken vigorously and left to stand at room temperature for 21 min in the dark. The absorbance was determined at 517 nm with a microplate reader. All samples were analyzed in triplicate and averaged.

The NO scavenging activity of brevinin-1FL was measured with the Griess reagent. In short, 2.5 mM sodium nitroprusside was incubated with brevinin-1FL (0-40 *μ*M) in a 96-well plate for 1 h at room temperature. NO generation was quantified with the Griess reagent, and the scavenging rate was calculated as the decrease in NO production compared to the group without brevinin-1FL treatment.

The reducing power of brevinin-1FL was evaluated with the ferric reducing antioxidant power (FRAP) method as previously reported by us with minor modification [11]. The activated FRAP working reagent (180 *μ*L) was loaded into a 96-well plate and incubated with 5 *μ*L of brevinin-1FL (0-40 *μ*M) or distilled water for 5 min at 37°C. The absorbance at 593 nm was measured with a microplate reader. Total antioxidant capacity was counted with the standard curve and expressed as the corresponding concentration of FeSO_4_ solution.

To study whether brevinin-1FL is a potent hydroxyl radical scavenger and suppresses hydroxyl radical-induced DNA damage, 2 *μ*L brevinin-1FL (20-320 *μ*M) was mixed with 0.5 *μ*g of pBR322 DNA dissolved in 6 *μ*L of 25 mM PBS (pH 7.4) containing 2 mM FeSO_4_ before being coincubated with 4 *μ*L of 30% H_2_O_2_ at 37°C for 60 min. 0.8% agarose gel electrophoresis was performed to examine DNA bands of the mixtures.

### 2.6. Cell Viability Measurement

PC12 cells were cultured in RPMI-1640 medium (Gibco, Chicago, USA) containing 10% fetal bovine serum (Gibco, Chicago, USA) and 100 U/mL penicillin-streptomycin at 37°C in a 5% CO_2_ incubator. Cell viability was determined using the MTT assay kit as previously reported by us with small modification [11]. In short, 1 × 10^4^ cells/well PC12 cells were plated in 96-well plates and grown overnight. Next, PC12 cells were incubated with H_2_O_2_ (0, 0.125, 0.5, and 1 mM) for 6 h or with brevinin-1FL (0-40 *μ*M) for 24 h or with brevinin-1FL (0, 2, 4, and 8 *μ*M) for 30 min before further coincubation with H_2_O_2_ for 6 h at 37°C, respectively. 10 *μ*L of MTT solution was loaded into each well and reincubated in the dark for 4 h. The culture supernatants were then carefully discarded, and 200 *μ*L of DMSO was applied to dissolve the formazan crystals in each well. The absorbance at 570 nm was determined.

### 2.7. LDH Release Assay

The LDH release assay was carried out with the LDH kit (Beyotime, Shanghai, China) in light of the manufacturer's manual. In brief, PC12 cells were preincubated with brevinin-1FL (0, 2, 4, and 8 *μ*M) for 30 min prior to coincubation with H_2_O_2_ for another 6 h at 37°C. After centrifugation at 2,000 rpm for 5 min, 100 *μ*L of the supernatant was mixed with 60 *μ*L of substrate solution in the dark for 30 min, and the absorbance at 490 nm was determined with the microplate reader (Infinite M1000 Pro, Tecan Company, Switzerland). LDH release rate (%) = (*A*_sample_ − *A*_blank_)/(*A*_LDH Release reagent_ − *A*_blank_) × 100, where *A*_blank_ is the absorbance of cell-free culture medium and *A*_sample_ is the absorbance in supernatant of cells. All experiments were conducted in triplicate.

### 2.8. Cell Morphology Assessment

PC12 cells at a density of 5 × 10^5^ cells/well were plated to 6-well plates and allowed to grow overnight before being pretreated with brevinin-1FL (0, 2, 4, and 8 *μ*M) for 30 min prior to exposure to 0.25 mM H_2_O_2_ for 6 h. Morphological observation was carried out with an inverted phase contrast microscope (CKX41, Olympus, Tokyo, Japan) at 100× magnification. About 4-5 single-plane photographs per well were acquired.

### 2.9. Antioxidant Capacity Measurement

The SOD and CAT activities as well as GSH and MDA contents in PC12 cells treated with brevinin-1FL were measured to further identify its antioxidant capacities. In brief, 5 × 10^5^ cells/well PC12 were seeded into 6-well plates and allowed to grow overnight. Next, cells were incubated with brevinin-1FL (0, 2, 4, and 8 *μ*M) for 30 min prior to incubation with 0.25 mM H_2_O_2_ for another 6 h. After washing three times with cold PBS, the cells were harvested by centrifugation and lysed on ice. The supernatant was transferred to fresh tubes and stored on ice or frozen at -80°C before the measurement of MDA, SOD, CAT, and GSH using commercial assay kits (S0131, S0101, S0051, and S0052; Beyotime Institute of Biotechnology, Shanghai, China), respectively. The assay for SOD activity was based on the ability of SOD to inhibit water-soluble tetrazolium salt (WST-8) reduction by superoxide. Briefly, 20 *μ*L of the supernatant was mixed with 160 *μ*L of WST-8/enzyme working solution and 20 *μ*L reacting solution and then incubated at 37°C for 30 min. Finally, the absorbance at 450 nm was determined with the microplate reader. One unit of SOD activity was defined as the amount of protein showing a 50% inhibitory effect on WST-8. The CAT activity was detected by a chromogenic substrate method. Briefly, 10 *μ*L of the supernatant was treated with excess H_2_O_2_ for 5 min. The remaining H_2_O_2_ coupled with a chromogenic substrate was catalyzed with peroxidase to generate red N-4-antipyryl-3-chloro-5-sulfonate-p-benzoquinonemonoimine. The absorbance at 520 nm was detected by using a microplate reader. One unit of CAT activity is defined as the amount of enzyme catalyzing 1 *μ*M of H_2_O_2_ per mg per min at 25°C. The total GSH level was measured with the enzymatic recycling method based on the fact that GSH can be oxidized by 5,5-dithiobis-2-nitrobenzoic acid (DTNB) to generate yellow 2-nitro-5-thiobenzoic acid and reduced by NADPH in the presence of glutathione reductase. 10 *μ*L of the supernatant was mixed with 150 *μ*L of DNTB solution for 5 min at room temperature and then incubated with 50 *μ*L of reaction solution containing NADPH and glutathione reductase for another 5 min. Finally, GSH concentration can be determined by measuring the absorption at 412 nm. The intracellular level of GSH was calculated based on cellular protein concentration. The concentration of MDA which is an indicator of lipid peroxidation in cells was measured using the thiobarbituric acid (TBA) method. In brief, 100 *μ*L of the supernatant was mixed with 200 *μ*L of the TBA reagent in a boiling water bath for 15 min. After centrifugation at 2,000 rpm at room temperature for 10 min, the supernatant was measured at a wavelength of 530 nm under a microplate reader. MDA level unit was expressed as nmol/mg of protein. All experiments are repeated three times.

### 2.10. Cell Cycle and Apoptosis Measurement

The effects of brevinin-1FL on the cycle distribution and apoptosis of PC12 cells stimulated by H_2_O_2_ were measured with flow cytometry (BD FACSCanto II, MA, USA). In brief, PC12 cells were seeded into 6-well plates at the density of 5 × 10^5^ cells/well and allowed to grow overnight. The medium was discarded, and cells were subsequently incubated with brevinin-1FL (2, 4, and 8 *μ*M) for 30 min before 0.25 mM H_2_O_2_ was added. After cells fixed in cold 70% ethanol, the cells were collected by centrifugation and stained with propidium iodide and RNase (Beyotime, Shanghai, China) for 30 min at 37°C to measure the proportions of cell cycle distribution. For the apoptosis assay, after being grown in normal medium and treated with H_2_O_2_ and brevinin-1FL (2, 4, and 8 *μ*M) for 6 h, the cells were collected and stained with propidium iodide and Annexin V-FITC at room temperature for 30 min in the dark in light of the manufacturer's manual (Beyotime, Shanghai, China). All stained cells were analyzed by flow cytometry with a minimum of 10,000 cells. All experiments were performed in triplicate. The data were analyzed using FlowJo (ver. 7.6).

### 2.11. ROS Detection

To evaluate the effect of brevinin-1FL on ROS generation in PC12 cells, DCFH-DA was used to examine the ROS level in differentially treated PC12 cells in light of the manufacturer's manual (Sigma-Aldrich; Darmstadt, Germany). In brief, 5 × 10^5^ PC12 cells were incubated with brevinin-1FL (0, 2, 4, and 8 *μ*M) for 30 min before 0.25 mM H_2_O_2_ was added. After 6 h incubation, the cells were collected and gently washed with PBS. Then, cells were incubated with DCFH-DA for 30 min in the dark at 37°C, washed three times, and measured by flow cytometry (BD FACSCanto II, Mansfield, MA, USA). There were three samples in each group for the ROS detection assay.

### 2.12. Mitochondrial Membrane Potential (ΔΨ*m*) Assay

ΔΨ*m* was determined with the JC-1 detection kit according to the manufacturer's manual (Beyotime, Shanghai, China). Briefly, the treated cells were collected and incubated with 500 *μ*L JC-1 working solution at 37°C with 5% CO_2_ for 20 min. Finally, the cells were washed before observation under the fluorescence microscopy at 400× magnification, and about 5-10 single-plane images per randomly selected area were acquired.

### 2.13. Western Blot Analysis

Western blots were carried out according to the method reported previously by us with minor modification [11]. In brief, cells were harvested and lysed with RIPA lysis buffer containing protease and phosphatase inhibitors (FDbio, Hangzhou, China) at 4°C for 15 min to obtain the sample for western blot analysis. Primary antibodies against phospho-AKT, AKT, phospho-ERK, ERK, phospho-JNK, JNK, phospho-p38, p38, p65, Bax, Bcl-2, PARP, cleaved PARP, caspase 3, cleaved caspase 3, and *β*-actin (4°C, 16 h, 1 : 2000; Cell Signaling Technology, Massachusetts, USA) and HRP-conjugated secondary antibodies (26°C, 1 h, 1 : 2000; Cell Signaling Technology, Massachusetts, USA) were used for western blot analysis. The band densities were quantified by using ImageJ software, and all experiments were performed in triplicate. Cells treated with medium alone were used as the negative control.

### 2.14. Carrageenan-induced Paw Edema Assay

The anti-inflammatory and antioxidant activities were evaluated with the paw edema induced by carrageenan based on the method previously reported by us with minor modifications [12]. In short, the mice paw volume up to the ankle joint was measured with a plethysmometer (Taimeng PV-200 7500, Chengdu, China) as the baseline value before mice were given intraperitoneal injection with brevinin-1FL (10 mg/kg), saline, or indomethacin (10 mg/kg). After 1 h, the plantar side of the right hind paw was injected with 50 *μ*L of 1% carrageenan suspended in saline or saline to induce swelling and edema. The paw volume was then determined at 0, 1, 2, 3, 4, and 5 h with a plethysmometer. The animals were euthanized with an overdose of pentobarbital (200 mg/kg intraperitoneally) 5 hours after injection, and the right hind paws of all mice were surgically cut off to prepare sample for the measurement of inflammatory factors (IL-1*β*, IL-6, and TNF-*α*), oxidative stress-related indicators (SOD and CAT activity and GSH level as well as MDA content), and myeloperoxidase (MPO) activity and histological analysis.

### 2.15. Statistical Analysis

All experiments were repeated at least three times. All data were analyzed using the GraphPad Prism software version 5.03 (GraphPad Software, CA, USA) and expressed as the mean ± SD. One-way ANOVA with the Tukey multiple comparison posttest was used for multiple-group analysis. A value of *p* < 0.05 was considered to represent a statistically significant difference.

## 3. Results

### 3.1. Identification and Characterization of Brevinin-1FL

**Figure 1:**
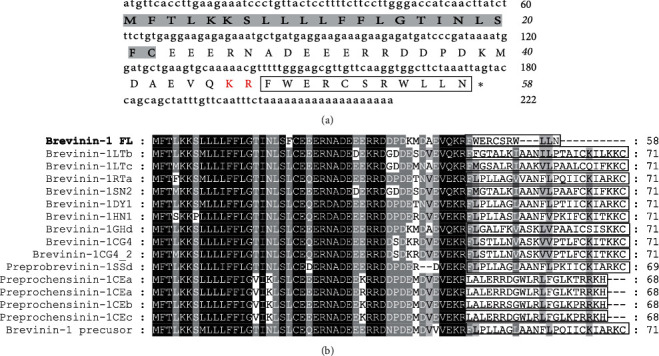
Sequence characterization of brevinin-1FL. (a) cDNA and deduced amino acid sequences of brevinin-1FL. The signal peptide is emphasized in gray, and the KR residues in red indicate the end of an acidic spacer domain. The sequence of mature peptide is boxed, and the stop codon is denoted by an asterisk (∗). Nucleotide and amino acid numbers are displayed after the sequences. (b) Multisequence alignment of brevinins-1 from frogs. In order to maximize structural similarity, residue deletions denoted by the hyphens (-) have been introduced in some sequences and the identical residues are displayed in black. The highly conserved residues are shaded. The domains of putative mature peptides are boxed.

The cDNA sequence encoding brevinin-1FL was cloned from the skin of *F. limnocharis* by the PCR-based method. As displayed in Figure 1, the cDNA of brevinin-1FL was 222 bp length and its deduced precursor contained 58 amino acid residues which possessed the classic sequence characteristic of amphibian defensive peptides, generally consisting of a predicted signal peptide sequence with 22 residues, an N-terminal acidic interval domain which was separated from the C-terminal mature peptide by a well-known KR protease cleavage site (Figure 1(a)). In light of the NCBI Basic Local Alignment Search Tool analysis, the deduced precursor shared high sequence identities to those of AMPs grouped into the brevinin-1 family. However, the amino acid sequence of its mature peptide named as brevinin-1FL, FWERCSRWLLN, lacked such similarity with any reported AMPs (Figure 1(b)). Brevinin-1FL had a theoretical PI of 9.24 with +3 net charge, and the aliphatic index was 104.33. Its relative mass was measured to 1509.82 Da.

### 3.2. Antioxidant Activity of Brevinin-1FL in Vitro

**Figure 2:**
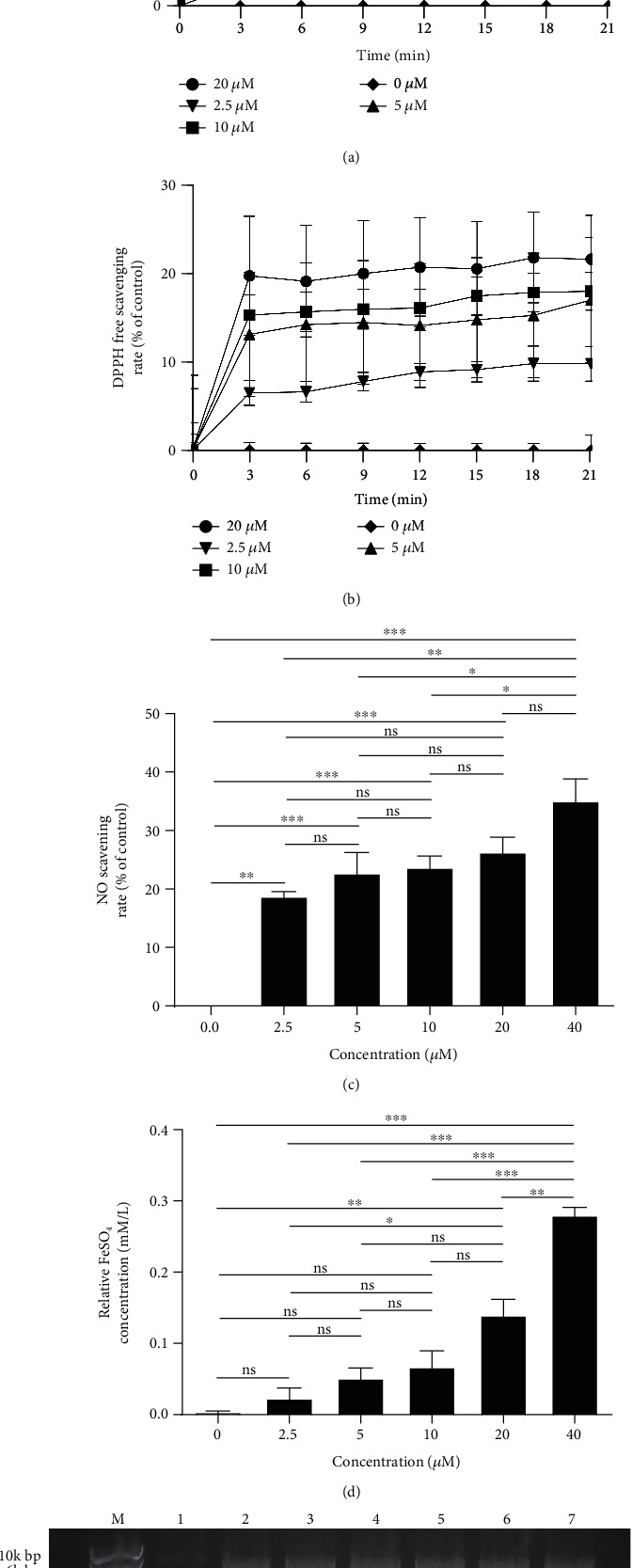
Antioxidant properties of brevinin-1FL *in vitro*. (a) ABTS radical scavenging activity. (b) DPPH radical scavenging activity. (c) NO scavenging activity. (d) FRAP antioxidant activity. (e) Hydroxyl radical scavenging activity. Lane M: marker. Lane 1: pBR322 (0.5 *μ*g) plasmid DNA control. pBR322 DNA was exposed to 2 mM FeSO_4_ plus 30% H_2_O_2_ in the absence (lane 2) or the presence of brevinin-1FL (320, 160, 80, 40, and 20 *μ*M) at 37°C for 1 h (lanes 3-7) before agarose gel electrophoresis of DNA and image capture, respectively. SC DNA: supercoiled DNA; OC DNA: open circular DNA. Data are presented as mean ± SD (*n* = 3). ns: no significant. ^∗^*p* < 0.05, ^∗∗^*p* < 0.01, and ^∗∗∗^*p* < 0.001 compared with each concentration.

ABTS and DPPH radical-scavenging assays were carried out to measure the antioxidant activity of brevinin-1FL. As illustrated in Figures 2(a) and 2(b), brevinin-1FL exhibited a dose-dependent ABTS and DPPH radical-scavenging power across the measured concentrations (0, 2.5, 5, 10, and 20 *μ*M). Brevinin-1FL eliminated approximately 64.20% of ABTS and 23.03% of DPPH at 21 min. NO, an important physiological mediator, has neurotoxic and proapoptotic effects when it is excessively generated [13]. As described in Figure 2(c), brevinin-1FL scavenged NO in a dose-dependent manner. The total antioxidant activity of brevinin-1FL was also examined with the FRAP method. As shown in Figure 2(d), the ferric reducing ability of brevinin-1FL was increased with increasing concentrations. In addition, the antioxidant activity of brevinin-1FL was also tested with the DNA protection assay. Hydroxyl radical produced from the Fenton reaction can induce a single-strand break into supercoiled plasmid DNA and final formation of open circular DNA after incubation with plasmid DNA. As shown in Figure 2(e), plasmid DNA incubated with Fe^2+^/H_2_O_2_ caused more formation of open circular DNA. However, brevinin-1FL concentration-dependently reduced this conversion of supercoiled DNA to the open circular DNA, suggesting that brevinin-1FL is a potent hydroxyl radical scavenger and can suppress the DNA damage induced by hydroxyl radical. All in all, these results demonstrate that brevinin-1FL is a potent and rapid antioxidant *in vitro*.

### 3.3. Internalization of Brevinin-1FL into PC12 Cells via Endocytosis

**Figure 3:**
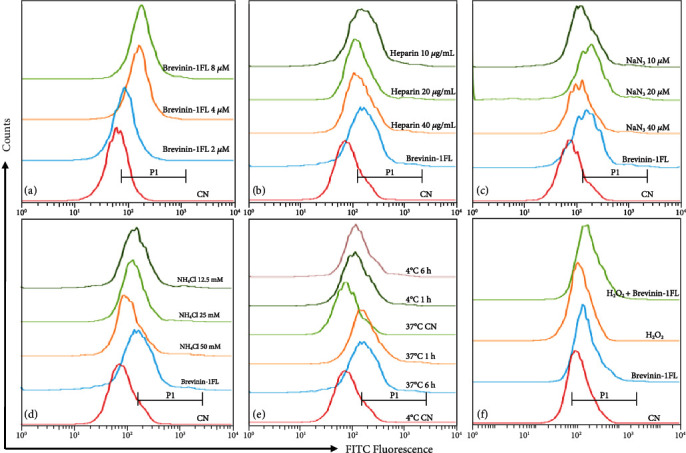
Flow cytometry analysis of brevinin-1FL internalized into PC12 cells under different conditions. Flow cytometry was carried out after PC12 cells were incubated with (a) FITC-labeled brevinin-1FL (2-8 *μ*M) or 8 *μ*M FITC-labeled brevinin-1FL plus (b) heparin (10-40 *μ*g/mL), (c) NaN_3_ (10-40 *μ*M), (d) NH_4_Cl (12.5-50 mM) for 6 h, and (e) 8 *μ*M FITC-labeled brevinin-1FL at the indicated temperature and time conditions or (f) 8 *μ*M FITC-labeled brevinin-1FL in the presence or absence of 0.25 mM H_2_O_2_ for 6 h at 37°C. CN: cells were treated with free FITC. The fluorophore shifts were compared using the half-offset style, and cells were gated according to FITC fluorescence (P1).

Compared with the control group, brevinin-1FL could be rapidly internalized into PC12 cells as shown by the increasing fluorescence in the cells treated with FITC-labeled brevinin-1FL in dose-dependent manners (Figure 3(a)). Anionic heparin sulfate is an important component in the cell membrane and the extracellular matrix associated with the first step of the interaction between the membrane and a cell-penetrating peptide [14]. Thus, we further investigated the effects of heparin sulfate on the cellular internalization of brevinin-1FL. As displayed in Figure 3(b), the uptake of 8 *μ*M FITC-labeled brevinin-1FL was reduced about 0.1%, 10.8%, and 15.6% after 1 h cotreatment with 10, 20, and 40 *μ*g/mL heparin, respectively. Next, we examined whether the internalization of brevinin-1FL required energy. Both NH_4_Cl and NaN_3_ are endocytic inhibitors because they can increase the pH of acidic endocytic vesicles and abolish ATP production within the cell membrane, respectively [15]. As shown in Figures 3(c) and 3(d), preincubation of PC12 cells with NaN_3_ and NH_4_Cl for 1 h obviously inhibited the cellular uptake of brevinin-1FL in concentration-dependent manners. As a further confirmation assay, the effect of temperature on the cellular endocytosis of brevinin-1FL was also examined. As shown in Figure 3(e), the cellular uptake of brevinin-1FL at 37°C was significantly higher than that at 4°C after 1 h or 6 h incubation, suggesting an energy-dependent and thermo-sensitive endocytosis of brevinin-1FL into PC12 cells (Figure S2). Notably, there was no obvious difference between different incubation times under the same temperature, indicating that brevinin-1FL can fast be internalized into PC12 cells. The influence of 0.25 mM H_2_O_2_ on the cellular penetration of brevinin-1FL was tested; coincubation with H_2_O_2_ in PC12 cells for 6 h could not significantly influence the cellular uptake of brevinin-1FL. All these results indicate that brevinin-1FL can be internalized into PC12 cells via endocytosis (Figure 3(f)).

### 3.4. Effect of Brevinin-1FL on Oxidative Stress-induced Cell Death

**Figure 4:**
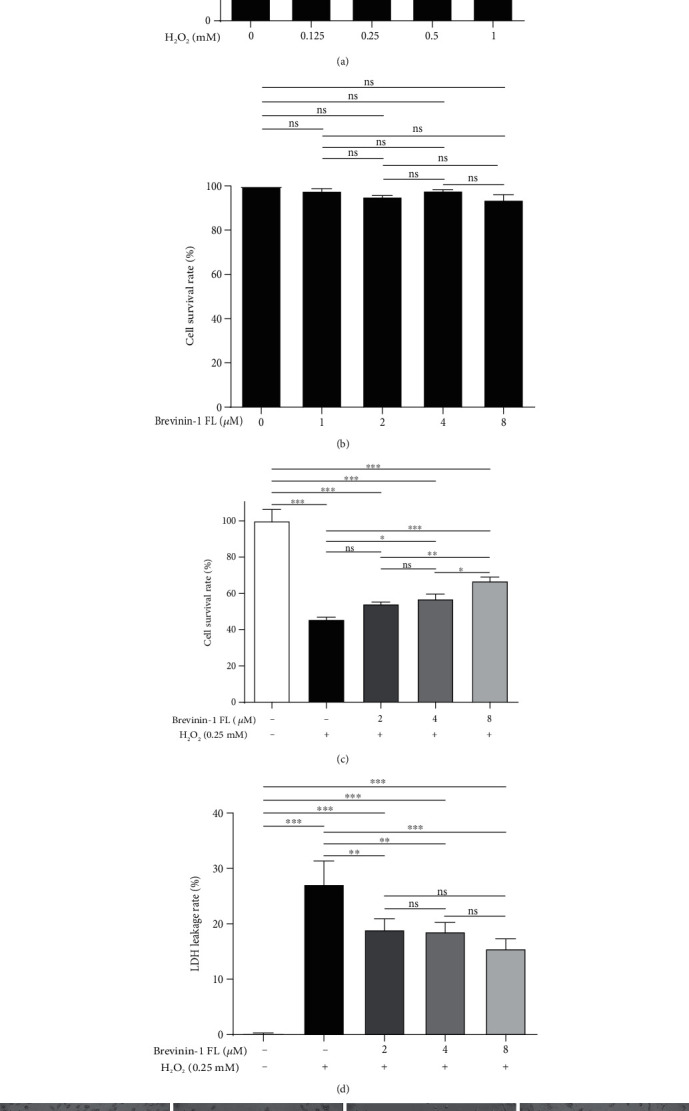
Protective effects of brevinin-1FL on PC12 cells stimulated by H_2_O_2_. (a–c) Relative viability of PC12 cells treated with H_2_O_2_, brevinin-1FL, and H_2_O_2_ plus brevinin-1FL. The cells were treated with the indicated concentrations of H_2_O_2_ for 6 h, brevinin-1FL for 24 h, or brevinin-1FL for 30 min and then with 0.25 mM H_2_O_2_ for 6 h before cell viabilities were measured by the MTT method. (d) Effects of brevinin-1FL on the LDH release of PC12 cells treated with H_2_O_2_. The PC12 cells were pretreated with brevinin-1FL for 30 min prior to coincubation with H_2_O_2_ for another 6 h before the LDH release was measured with a LDH kit. (e) Morphological observation of PC12 cells. (a) is the control without treatment. (b)–(d) are typical images of cells treated with H_2_O_2_, H_2_O_2_+4 *μ*M brevinin-1FL, and H_2_O_2_+8 *μ*M brevinin-1FL, respectively. Magnification: 100x. Data are shown as the percentage of the control group (mean ± SD; *n* = 3). ns: not significant. ^∗^*p* < 0.05, ^∗∗^*p* < 0.01, and ^∗∗∗^*p* < 0.001 compared with each group.

The cytotoxicity of a series of concentrations of H_2_O_2_ towards PC12 cells was measured to ensure the protective effect of brevinin-1FL on oxidative stress-induced cell death. As shown by MTT results in Figure 4(a), H_2_O_2_ could concentration-dependently decrease the viability after 6 h treatment and the viability of PC12 cells treated with 0.25 mM H_2_O_2_ significantly reduced to about 48.35%. However, pretreatment with 2, 4, and 8 *μ*M of brevinin-1FL which has no toxic effect on PC12 cells increased cell viability by 55.17%, 57.23%, and 65.61% when compared to control cells treated with 0.25 mM H_2_O_2_ (Figures 4(b) and 4(c)). Lactate dehydrogenase (LDH) release was investigated to evaluate the protective mechanism of brevinin-1FL against oxidative stress-induced cell death. As shown in Figure 4(d), brevinin-1FL reduced the LDH release of H_2_O_2_-treated cells in a dose-dependent manner. Notably, after PC12 cells were treated with 0.25 mM H_2_O_2_ for 6 h, the discrepancy between the proliferation inhibition rate and the inhibition rate of LDH release indicating other mechanisms exists in cell death induced by H_2_O_2_ except necrosis (Figures 4(c) and 4(d)). In line with the MTT results, morphological observation of PC12 cells showed that the unstimulated cells grew better than H_2_O_2_-treated cells, with a dendritic shape and even distribution while treatment with brevinin-1FL reversed these features (Figure 4(e)).

### 3.5. Effect of Brevinin-1FL on Intracellular SOD and CAT Activity and MDA and GSH Content

**Figure 5:**
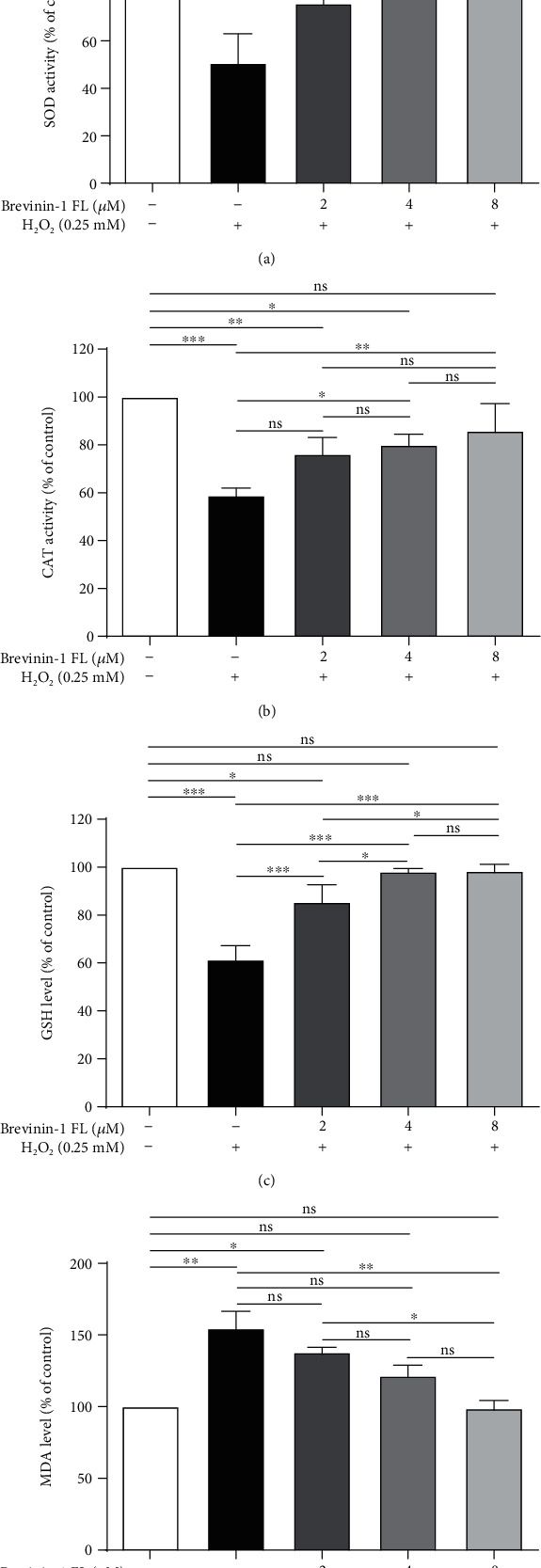
Suppression of brevinin-1FL to oxidative damage in H_2_O_2_-induced PC12 cells. PC12 cells were preincubated with brevinin-1FL for 30 min and then incubated with H_2_O_2_ for 6 h before the activity of SOD (a) and CAT (b); also, the contents of GSH (c) and MDA (d) were measured via spectrophotometry. Data are shown as the percentage of the control group (mean ± SD; *n* = 3). ns: not significant. ^∗^*p* < 0.05, ^∗∗^*p* < 0.01, and ^∗∗∗^*p* < 0.001 compared with each group.

MDA is a peroxidation product under oxidative stress while SOD, CAT, and GSH are antioxidants in cells. They are generally applied in the research of antioxidant drugs as pharmacodynamic indicators [1]. When compared with the control group, three antioxidant contents in H_2_O_2_-treated PC12 cells were restored by brevinin-1FL in concentration-dependent manners (Figures 5(a)–5(c)). Consistently, MDA levels were significantly increased by approximately 164.64% in PC12 cells treated with 0.25 mM H_2_O_2_. However, this increase was significantly inhibited by about 15.01%, 19.47%, and 28.13% after the cells were treated with 2, 4, and 8 *μ*M of brevinin-1FL, respectively (Figure 5(d)).

### 3.6. Effect of Brevinin-1FL on H_2_O_2_-induced ROS Production and Mitochondrial Membrane Potential

**Figure 6:**
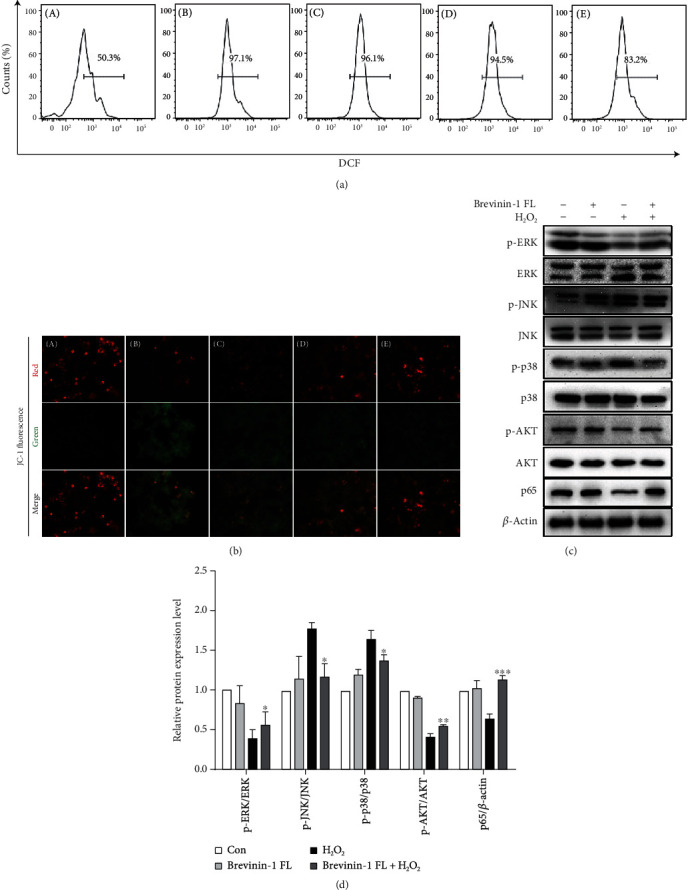
Regulation of ROS generation, mitochondrial damage, and AKT/MAPK/NF-*κ*B signaling cascades by brevinin-1FL in H_2_O_2_-treated PC12 cells. PC12 cells were preincubated with brevinin-1FL (2, 4, and 8 *μ*M) for 30 min and then incubated with H_2_O_2_ for another 6 h before (a) ROS levels were analyzed by flow cytometry and (b) mitochondrial Δ*ψm* was detected by fluorescence microscopy. A–E in (a) and (b) are from the control group without treatment, H_2_O_2_ treatment group, and 2, 4, and 8 *μ*M of brevinin-1 pretreatment groups, respectively. DCF: the fluorescence level of dichlorofluorescein which transformed from DCFH-DA in cells. (c) Representative western blot images of proteins in MAPK, NF-*κ*B, and AKT pathways. (d) Statistical analysis of quantified band densities in western blots. Bars represent the relative expression of the target protein to the control. Band densities were analyzed by ImageJ software, and data are mean ± SD (*n* = 3). ^∗^*p* < 0.05, ^∗∗^*p* < 0.01, and ^∗∗∗^*p* < 0.001 compared with the H_2_O_2_-induced group.

ROS play the critical role in the regulation of cell proliferation as well as survival and mediate oxidative damage to lipids, DNA, and proteins, representing a key pathogenic role in neurodegenerative diseases [1, 2]. Hence, the effect of brevinin-1FL on H_2_O_2_-induced total intracellular ROS accumulation in PC12 cells was examined. As shown in Figure 6(a), ROS contents were evidently increased after H_2_O_2_ treatment. But this increase was significantly inhibited by pretreatment with brevinin-1FL in a concentration-dependent manner. The ROS production is closely associated with the mitochondrial membrane potential (Δ*ψm*) which can be used to evaluate mitochondrial function and test the protective effect of antioxidant peptides on cells suffering from oxidative stress. As shown by the fluorescence in Figure 6(b), the red fluorescence indicating the intact mitochondrial membrane was obviously detectable in all cells, and H_2_O_2_ stimulation for 6 h resulted in an evident fluorescence shift from red to green, suggesting a decrease in Δ*ψm* and damage to the mitochondrial membrane. However, pretreatment with 2, 4, and 8 *μ*M of brevinin-1FL concentration-dependently reversed this effect induced by H_2_O_2_ and increased the red fluorescence, suggesting a protective effect of brevinin-1FL on H_2_O_2_-induced damage to the mitochondrial membrane.

ROS can adjust AKT/MAPK/NF-*κ*B signaling pathways which are involved in apoptosis and inflammation responses [1, 2]. Therefore, western blot was carried out to examine the effects of brevinin-1FL on their activation in H_2_O_2_-induced PC12 cells. As displayed in Figures 6(c) and 6(d), after exposure to 0.25 mM H_2_O_2_ for 6 h, the expression of phosphorylated p38 and JNK was obviously upregulated in PC12 cells. In contrast, the contents of p65 as well as phosphorylated ERK and AKT in the cytoplasm were significantly downregulated, which suggest that AKT/MAPK/NF-*κ*B pathways are responsible for H_2_O_2_-induced apoptosis in PC12 cells. Nevertheless, brevinin-1FL reversed the changes induced by H_2_O_2_, demonstrating that the protective effects of brevinin-1FL in PC12 cells treated with H_2_O_2_ are associated with its regulation of AKT/MAPK/NF-*κ*B signaling pathways.

### 3.7. Effect of Brevinin-1FL on Apoptosis and Cell Cycle Arrest

**Figure 7:**
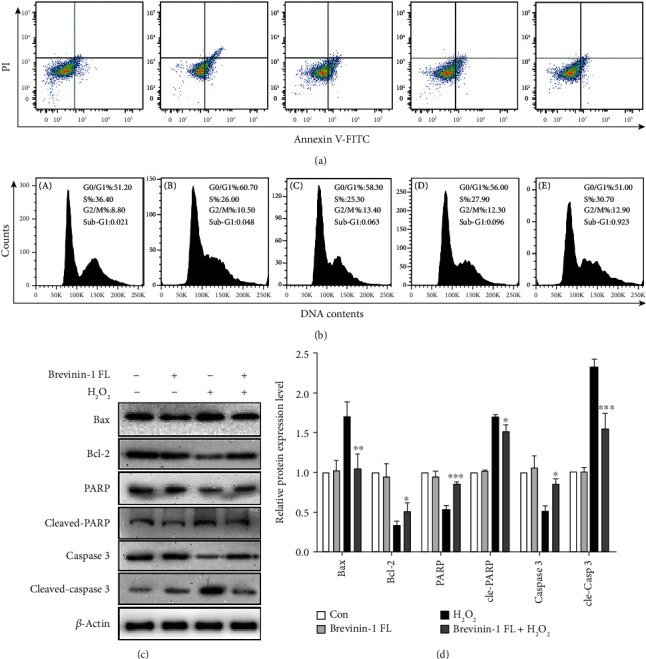
Inhibition of H_2_O_2_-induced apoptosis and cycle arrest by brevinin-1FL in PC12 cells. PC12 cells were pretreated with different concentrations of brevinin-1FL for 30 min and then incubated with H_2_O_2_ for another 6 h before cell cycle and apoptosis were analyzed with flow cytometry. (a) Cell apoptosis analysis. The percentage is the ratios of normal, early, and late apoptotic and necrotic cells. (b) Cell cycle analysis. A–E in (a) and (b) are from the control group without treatment, H_2_O_2_ treatment group, and 2, 4, and 8 *μ*M of brevinin-1 pretreatment groups, respectively. (c) Western blot analysis of Bax, Bcl-2, PARP, cleaved PARP, caspase 3, and cleaved caspase 3 in H_2_O_2_-induced PC12 cells. (d) Statistical analysis of quantified band densities of the brevinin-1FL-treated group compared with the H_2_O_2_-induced group. Bars represent the relative expression of the target protein to the control. Band densities were analyzed by ImageJ software, and data are mean ± SD (*n* = 3). ^∗^*p* < 0.05, ^∗∗^*p* < 0.01, and ^∗∗∗^*p* < 0.001 compared with the H_2_O_2_-induced group.

Excessive ROS generation can cause cell cycle arrest and apoptosis due to its damage to mitochondrial functions in PC12 cells [11, 16]. As displayed in Figure 7(a), the number of apoptotic cells was obviously increased in H_2_O_2_-treated cells when compared to the control. But it was reduced by brevinin-1FL in a concentration-dependent manner, and the apoptotic inhibition rates in the presence of brevinin-1FL at 2, 4, and 8 *μ*M were around 24.30%, 35.23%, and 70.15%, respectively. Furtherly, H_2_O_2_ markedly augmented the cell numbers accumulating in the G0/G1 phase, which was accompanied by the reduced cell numbers in the S phase in PC12 cells, whereas brevinin-1FL concentration-dependently ameliorated this effect induced by H_2_O_2_ (Figure 7(b)). In agreement, exposure to H_2_O_2_ for 6 h resulted in an obvious expression increase in Bax and decrease in Bcl-2. However, the addition of 8 *μ*M brevinin-1FL to cells significantly counteracted these changes induced by H_2_O_2_. At the same time, brevinin-1FL significantly reduced PARP and caspase 3 cleavage upregulated by H_2_O_2_ (Figures 7(c) and 7(d)). Overall, the present data prove that brevinin-1FL effectively inhibits the H_2_O_2_-induced cell cycle arrest and apoptosis, consequently increasing the viabilities of PC12 cells.

### 3.8. Antioxidant and Anti-Inflammatory Activity of Brevinin-1FL in Vivo

**Figure 8:**
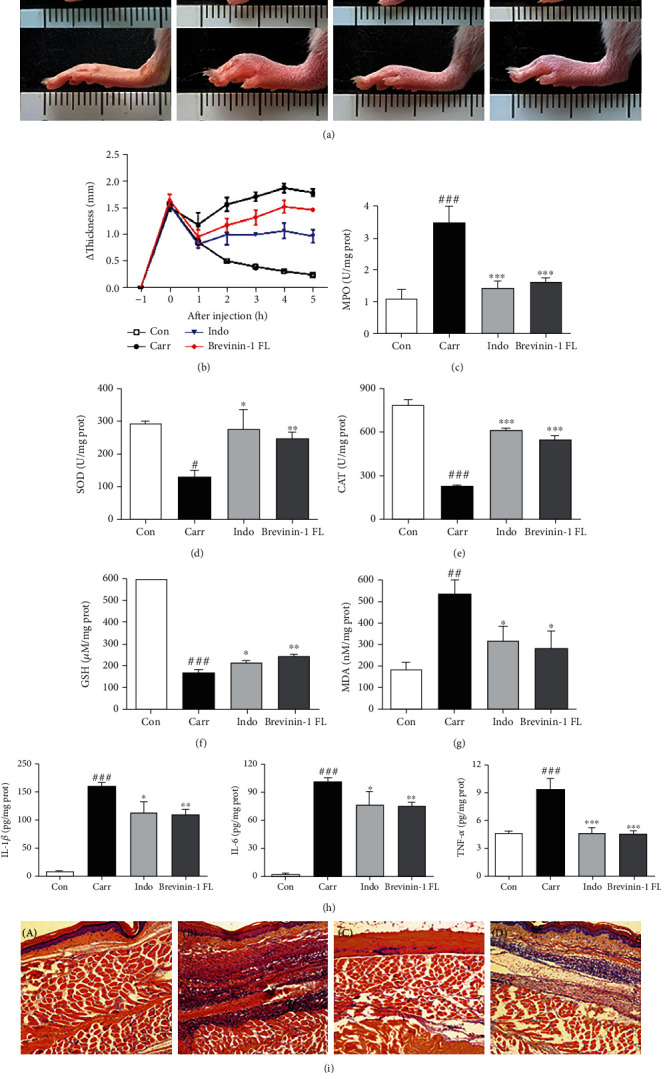
Antioxidant and anti-inflammatory activity of brevinin-1FL in mice. The paw edema was injected in their right hind pad by 50 *μ*L of 1% carrageenan 1 h after intraperitoneal administration of 10 mg/kg of brevinin-1FL or indomethacin or equal volume of saline. (a) Photographic images of mouse paws at 5 h postinjection. The upper and lower rows are the bottom and lateral views of paws, respectively. The unit of scale bars shown in images is centimeter. (b) Paw swelling degree at each time point after administration of carrageenan. (c) MPO activity in the mouse paws. Effects of brevinin-1FL on SOD (d) and CAT (e) activity and GSH (f) and MDA (g) contents as well as proinflammatory cytokine expression (h) in the paws injected by carrageenan. Statistical results are expressed as the mean ± SD (*n* = 6). ^#^*p* < 0.05, ^##^*p* < 0.01, and ^###^*p* < 0.001 compared with the control group. ^∗^*p* < 0.05, ^∗∗^*p* < 0.01, and ^∗∗∗^*p* < 0.001 indicate a significant difference compared with the carrageenan group. (i) HE staining images of carrageenan-induced paw edema at 5 h postinjection. A–D in (a) and (i) are the paws from mice treated with saline (a), carrageenan+saline (b), carrageenan+indomethacin (c), and carrageenan+brevinin-1FL (d), respectively.

Carrageenan-induced acute inflammation is known to be related to the accumulation of ROS, lipid peroxidation, and the impediment of antioxidant defense activities [17, 18]. We therefore assessed the anti-inflammatory and antioxidant ability of brevinin-1FL in the mouse paws injected by carrageenan as previously reported by us [12]. As illustrated in Figures 8(a) and 8(b), the volumes of paw edema were markedly increased by 1.81-fold after carrageenan injection in comparison with the control group. However, brevinin-1FL alleviated the paw swelling induced by carrageenan. In agreement, treatment with brevinin-1FL also suppressed the activity of MPO which is the indicator of neutrophil migration in carrageenan-injected paw tissues (Figure 8(c)). MDA is a mediator of inflammatory processes and a marker of cellular injury triggered by ROS and oxidative stress while endogenous antioxidant enzymes like SOD and CAT as well as GSH greatly contribute to eradicating the damaging effects of ROS and oxidative stress [19]. Carrageenan administration abated cellular SOD and CAT activity and GSH level and increased MDA concentration when compared to the control (Figures 8(d)–8(g)). The expression changes of IL-1*β*, TNF-*α*, and IL-6 were examined in serum and tissue of mice after carrageenan administration. As shown in Figure 8(h) and Figure S3, the above raised trends in the carrageenan-injected mice were reversed by brevinin-1FL and indomethacin. Consistently, histopathology analysis displayed that brevinin-1FL significantly weakened carrageenan-induced leukocyte infiltration (Figure 8(i)). Notably, brevinin-1FL (10 mg/kg) compared to indomethacin possessed greater inhibition effect on the increase in MDA contents induced by carrageenan. Together, these data underscore that brevinin-1FL possesses the antioxidant and anti-inflammatory activities.

## 4. Discussion

Oxidative stress, the overproduction of ROS in cells and tissues, reflects an imbalance between antioxidants and free radicals and leads to the damage of cellular biomacromolecules such as DNA, proteins, and lipids, consequentially causing many human health disorders including neurological illness, inflammation, diabetes, cancer, and atherosclerosis [1, 2]. Hence, natural and synthetic antioxidants protecting normal cells from damage derived from oxidants may have therapeutic potential and are increasingly recognized as a pivotal direction for the prevention and treatment of oxidation-associated diseases [1, 3, 5]. Some bioactive peptides identified from animals, especially from amphibian skin, have been found to display antioxidant and anti-inflammatory activities [5, 6]. However, there is no antioxidant peptide identified from *F. limnocharis.* Here, for the first time, we identify an antioxidant peptide from this tropical frog and explore its antioxidant effects plus underlying mechanism in H_2_O_2_-treated PC12 cells and carrageenan-stimulated mice paws.

Many antioxidant peptides containing different structures have been identified from amphibians, and the presence of critical residues of proline, leucine, phenylalanine, methionine, free cysteine, tyrosine, or tryptophan is responsible for their antioxidant activity [20–26]. In particular, cysteine containing the reducing thiol group provides more potent antioxidant capability than the above other amino acids to an antioxidant [27]. For brevinin-1FL, 1 free cysteine and 5 hydrophobic amino acids (2 leucine, 2 tyrosine, and 1 phenylalanine residues) are contained in its primary sequence (Figure 1(a)). Comparison with cathelicidin-OA1, antioxidin-I, and antioxidin-RL, brevinin-1FL contained higher proportion of hydrophobic amino acids and stronger antioxidant activity according to their radical-scavenging capability or protective effects on the cells with oxidative damage *in vitro* [21, 28, 29]. This result further demonstrates that the sequence of peptides has great effects on their antioxidant activity. It is a remarkable fact that the evolution and formation of antioxidant peptides in amphibians may be associated with long time exposure to sunshine and intense ultraviolet radiation [22, 24]. The frog *F. limnocharis* captured from tropical Guangdong Province live in low altitude (23.12°N, 113.28°E) with long and strong sunlight radiation. Besides, these frogs generally live near pond ditches with few protections from sunlight, making them easy to receive sunlight radiation [30]. Thus, the present study supports the conclusion that the evolution of antioxidant peptides is associated with sunlight radiation as reported by other researchers [22, 31, 32]. Some brevinin-1-like peptides from the frog skin secretions like LFB, brevinin-1OS, and their N-terminal derivatives show potent antimicrobial activities [33, 34]. Although it shares similar precursor structure with these peptides consisting of a signal peptide at the N-terminus followed by an acidic spacer region and a mature peptide at the C-terminus, brevinin-1FL like antioxidin-I and salamandrin-I does not show antibacterial activity against *S. aureus* ATCC 25923, *E. coli ATCC* 25922, and *P. aeruginosa* ATCC 27853 (data not shown) [24, 35]. What is more, mature brevinin-1FL lacks structural similarity with any reported AMP which is consistent with our experimental antibacterial results and further proves the conclusion that a balance between hydrophobicity, positive charge, and degrees of *α*-helicity is crucial for keeping antimicrobial activity of peptide (Figure 1(b)) [34]. Thus, the presence of antioxidant peptide in this frog skin might reflect adaptation to the specific environment [36].

Antioxidants often keep homeostasis and prevent cells and tissues from oxidative stress-induced disorders by removing excessive free radicals [1, 22]. MDA is a cell-damaging peroxidation product of biomacromolecules on the surface of cell membranes caused by oxidative stress and can gradually lead to damage and dysfunction of all intracellular protein functions [37]. The hydroxyl group is thought as a DNA-damaging agent of physiological significance and can cause carcinogenesis or characteristic in the pathogenesis of neurodegenerative diseases like Alzheimer's and Parkinson's disease [3]. SOD, CAT, and GST are the key endogenous antioxidants which can reduce the contents of the oxidants and provide a first line of defense against their potentially damaging effects [19, 38]. In this study, brevinin-1 FL has ability to scavenge free radicals like NO, hydroxyl radicals, ABTS^+^, and DPPH and reduce Fe^3+^*in vitro* (Figure 2), suggesting that it is an antioxidant peptide. The nervous system has high oxygen utilization, large amount of polyunsaturated fatty acids, and low contents of antioxidants, which make it very susceptible to oxidative assaults [3]. The PC12 cell line is particularly vulnerable to changes in O_2_ concentration and usually used as a cellular model to research neuronal sensitivity to oxidative stress [39]. At present study, the rat differentiated PC12 cells are subjected to H_2_O_2_ exposure for 6 h to mimic a neuronal *in vitro* model of oxidative injury. In agreement, H_2_O_2_ significantly decreases the viability of PC12 cells via increasing ROS accumulation and MDA contents and decreasing the levels of endogenous antioxidants and Δ*ψm* (Figures 5 and 6), which suggest the neuronal sensitivity to oxidative damage. However, brevinin-1FL can be internalized into PC12 cells via endocytosis (Figure 3) and successfully reverse the intracellular effects induced by H_2_O_2_, consequently decreasing the cycle arrest, apoptosis, and necrosis of PC12 cells caused by oxidative stress (Figures 4(d) and 7). It is generally accepted that the *in vitro* results are not able to assess cell-to-cell interactions and related issues of bioavailability, dose, and bioeffective concentrations of drugs *in vivo* [40]. Further, the injection of carrageenan can induce edema formation, free radical production, and secretion of proinflammatory cytokines like TNF-*α* and IL-1*β* because of the complex crosstalk between inflammation and ROS accumulation. As a result, carrageenan-induced paw edema is usually used to clarify the anti-inflammatory and antioxidant activity of some antioxidants *in vivo* [17, 18]. Here, 5 h after paw administration, carrageenan obviously increases MDA contents while it decreases the levels of endogenous antioxidants which play key roles to counteract inflammatory stress and oxidative damage caused by ROS [38]. In line with its *in vitro* results, brevinin-1FL significantly suppresses the above changes induced by carrageenan in paws (Figure 8). Moreover, in agreement with facts that ROS can affect expression of inflammatory cytokines and antioxidants can reduce the generation of the latter [17, 41, 42], brevinin-1FL inhibits paw edema, neutrophil infiltration, and the generation of proinflammatory IL-6, TNF-*α*, and IL-1*β* due to its antioxidant function in a mouse paw injected by carrageenan (Figure 8).

It is well known that excessive ROS accumulation contributes to mitochondrial damage in a range of pathologies and induces dissipation or even loss of Δ*ψm*, initiating to activate the mitochondria-mediated signal cascades [43, 44]. In this study, brevinin-1FL significantly increases Δ*ψm* and the Bcl-2/Bax ratio which play critical roles in mitochondria-mediated cell death [45, 46] and consequently decreases apoptosis as well as cycle arrest of PC12 cells with H_2_O_2_ treatment (Figures 6 and 7). Therefore, brevinin-1FL protects PC12 cells from H_2_O_2_-induced apoptosis and cycle arrest via regulating intracellular ROS levels and mitochondrial pathways. Increasing evidences also suggest that activation of JNK and p38 signaling pathways induced by oxidative stress can contribute to apoptosis and cell cycle arrest [47, 48]. NF-*κ*B, the target of phosphorylated p38, can be activated by oxidative stress which be inhibited by some antioxidants [49, 50], with the result of the decreased translocation of NF-*κ*B subunit p65 to the nucleus from the cytoplasm [11]. Further, it has been acknowledged that activated AKT and ERK are closely related to apoptosis suppression [51, 52]. In this study, H_2_O_2_ inactivates AKT and ERK, while it activates JNK, p38, and NF-*κ*B in PC12 cells (Figure 6), suggesting that these signal proteins exert an important role in the process of H_2_O_2_-induced abnormal apoptosis and cell cycle accumulation in PC12 cells [53, 54]. However, brevinin-1FL significantly reverses the above H_2_O_2_-induced changes after incubation with PC12 cell for 6 h, suggesting that its neuroprotective effects may be involved in the regulation of AKT/MAPK/NF-*κ*B signaling cascades which are closely associated with its antioxidant activity. It is common that antioxidants can exert antioxidant effects via activating the Nrf2 pathway while suppressing the components belonging to the MAPK pathway in different cells [50]. For example, ECM has been reported to restore the levels of cellular antioxidants including SOD and accordingly inhibit oxidative stress via reducing the phosphorylation of p38 and JNK while increasing the activation of ERK and Nrf2 in tBHP-induced PC12 cells and a D-gal plus AlCl_3_-induced neurodegenerative mouse model [16]. In addition, the activation of Nrf2 can be modulated by some kinases such as protein kinase C, ERK, and AKT [50]. At present study, brevinin-1FL increases the levels of the downstream antioxidant effectors (SOD, CAT, and GSH) in the Nrf2 pathway and restores the activation of ERK as well as AKT in PC12 cells (Figure 5). Additionally, brevinin-1FL possesses antioxidant protection effects and anti-inflammatory ability in carrageenan-induced paw edema. Therefore, we conclude that brevinin-1FL inhibits oxidative stress via the regulation of AKT/MAPK/NF-*κ*B and Nrf2 signaling pathways *in vitro* and *in vivo*.

## 5. Conclusion

We first identify brevinin-1FL, an antioxidant peptide from the frog *F. limnocharis*. Brevinin-1FL rapidly exhibits high reducing power and radical scavenging activities *in vitro*. Moreover, brevinin-1FL obviously rescues the viability and inhibits cycle arrest in the G0/G1 phase as well as apoptosis in H_2_O_2_-treated PC12 cells by restoring the intracellular levels of GSH, SOD, and CAT, decreasing the contents of MDA, causing the inhibition of ROS generation, increase of Δ*ψm*, and regulation of AKT/MAPK/NF-*κ*B signaling pathways under oxidative stress condition. Furthermore, brevinin-1FL shows strong antioxidant and anti-inflammatory activities by decreasing proinflammatory cytokine secretions as well as MPO and MDA contents and enhancing the levels of antioxidant enzymes in carrageenan-stimulated mice. To the best of our knowledge, this is the first systemic report about an antioxidant peptide derived from frog *F. limnocharis* skin. This study will help us to further comprehend the biological functions of amphibian skin and the mechanism by which antioxidants protect cells from oxidative stress and provide a candidate molecule with therapeutic potential for diseases induced by oxidative damage.

## Data Availability

The data used to support the findings of this study are available from the corresponding authors upon request.

## Abbreviations

H_2_O_2_: Hydrogen peroxide
ROS: Reactive oxygen species
NCBI: North Carolina Banking Institute
FITC: Fluorescein isothiocyanate
MTT: 3-(4,5-Dimethylthiazol-2-yl)-2,5-diphenyltetrazolium bromide
LDH: Lactate dehydrogenase
ABTS^+^: 2,2-Azinobis- (3-ethylbenzothiazoline-6-sulfonate)
DPPH: 2,2-Diphenyl-1-picrylhydrazyl
NO: Nitric oxide
FRAP: Ferric reducing antioxidant power
SOD: Superoxide dismutases
CAT: Catalases
GSH: Glutathione
MDA: Malondialdehyde
MAPK: Mitogen-activated protein kinase
NF-*κ*B: Nuclear factor kappa-B
ERK: Extracellular signal-regulated kinases
JNK: c-Jun N-terminal kinase
IL-1*β*: Interleukin-1 beta
IL-6: Interleukin-6
TNF-*α*: Tumour necrosis factor alpha
MPO: Myeloperoxidase
AMPs: Antimicrobial peptides.

## Supplementary Materials

Supplementary Materials Graphical abstract of the manuscript displayed in supplementary files. Supplement Figure S1: the characterization of synthesized brevinin-1FL. (A) RP-HPLC. (B) MALDI-TOF-MS. Figure S2: the statistical analysis of brevinin-1FL internalized into PC12 cells under different conditions by flow cytometry. Figure S3: effects of brevinin-1FL on the expression of IL-1*β*, TNF-*α*, and IL-6 in serum. Data presented are mean ± SD (*n* = 3). ^#^*p* < 0.05, ^##^*p* < 0.01, and ^###^*p* < 0.001 compared to the control group; ^∗^*p* < 0.05, ^∗∗^*p* < 0.01, and ^∗∗∗^*p* < 0.001 compared to the model group.
