# Supplementary material for: Multiple Mechanistic Action of Brevinin-1FL Peptide against Oxidative Stress Effects in an Acute Inflammatory Model of Carrageenan-Induced Damage
Source: Oxid Med Cell Longev. 2022 Sep 5;2022:2615178. doi: 10.1155/2022/2615178 (PMC9467757; doi:10.1155/2022/2615178)
Supplement: Supplementary Materials — Graphical abstract of the manuscript displayed in supplementary files. Supplement Figure S1: the characterization of synthesized brevinin-1FL. (A) RP-HPLC. (B) MALDI-TOF-MS. Figure S2: the statistical analysis of brevinin-1FL internalized into PC12 cells under different conditions by flow cytometry. Figure S3: effects of brevinin-1FL on the expression of IL-1β, TNF-α, and IL-6 in serum. Data presented are mean ± SD (n = 3). #p < 0.05, ##p < 0.01, and ###p < 0.001 compared to the control group; ∗p < 0.05, ∗∗p < 0.01, and ∗∗∗p < 0.001 compared to the model group. [file 2615178.f1.docx]

**Multiple mechanistic action of Brevinin-1FL peptide against oxidative stress effects in an acute inflammatory model of carrageenan induced damage**

Jinwei Chai^1,2^, Junfang Liu^1^, Maolin Tian^2^, Hang Liao^2^, Jiena Wu^2^, Jianpeng Xie^1,2^, Shian Lai^3^, Guoxiang Mo^4^, Xin Chen^1,^*, Xueqing Xu^2,^*

^1^Department of Pulmonary and Critical Care Medicine, Zhujiang Hospital, Southern Medical University, Guangzhou, China

^2^Guangdong Provincial Key Laboratory of New Drug Screening, School of Pharmaceutical Sciences, Southern Medical University, Guangzhou, China

^3^Department of Molecular Chemistry and Biochemistry, Faculty of Science and Engineering, Doshisha University, Kyotanabe, Japan.

^4^College of Life Sciences, Nanjing Agricultural University, Nanjing, China

Correspondence should be addressed to Xin Chen; chen_xin1020@163.com and Xueqing Xu; xu2003@smu.edu.cn

**Graphical Abstract**


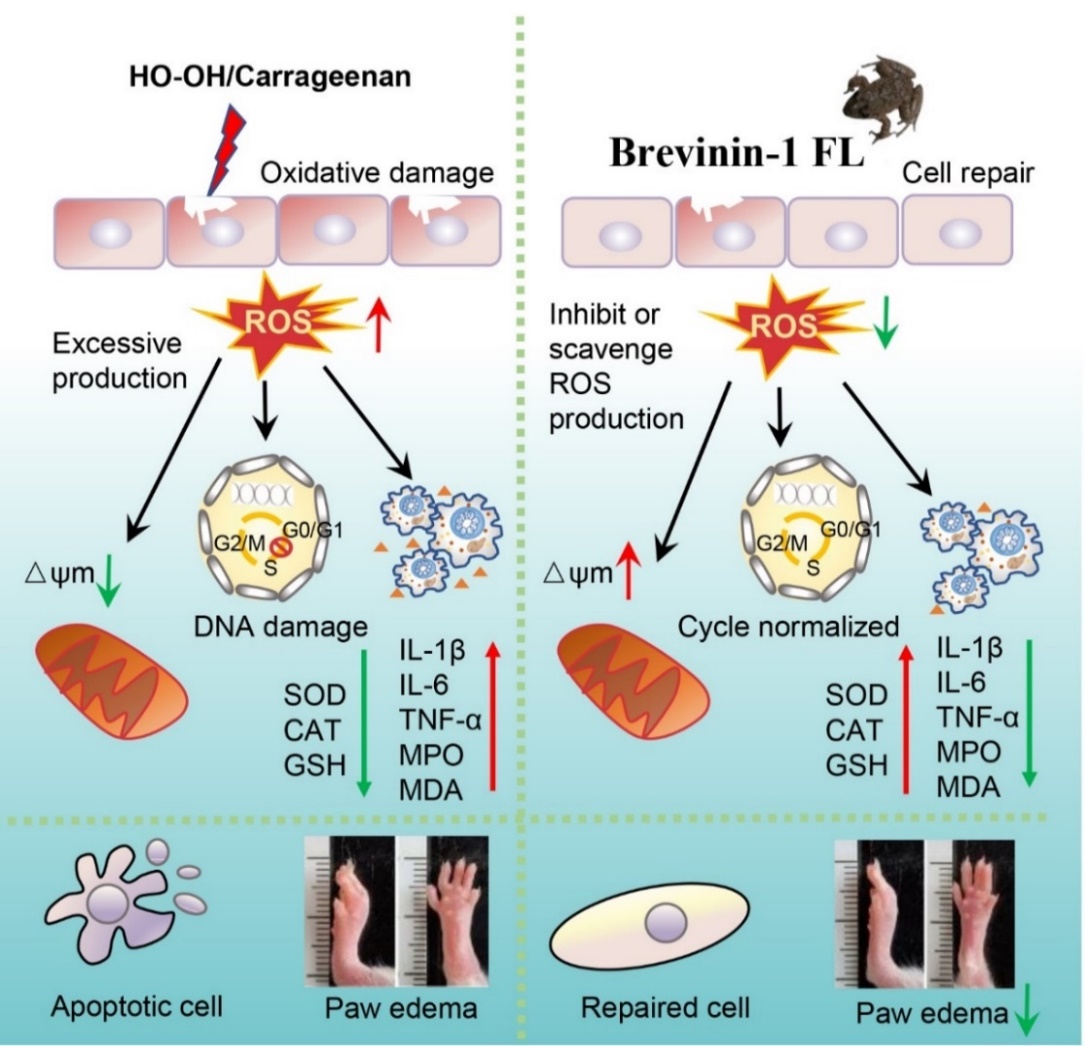


**Contents:**

**Supplementary Figures 1**

**Supplementary Figures 2**

**Supplementary Figures 3**

**Supplementary Figures**


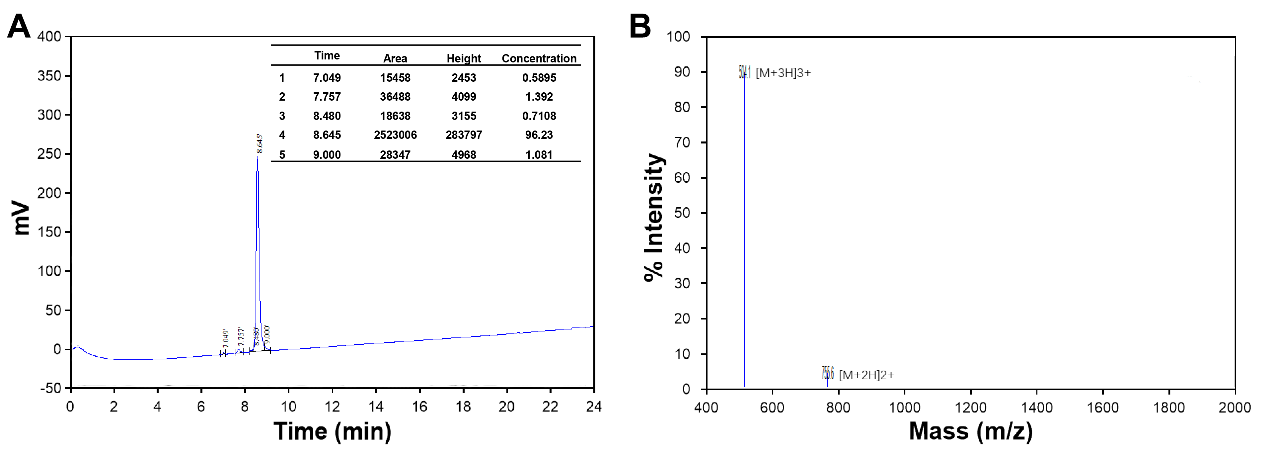


FIGURE S1: The characterization of synthesized Brevinin-1FL. (A) RP-HPLC. (B) MALDI-TOF-MS.


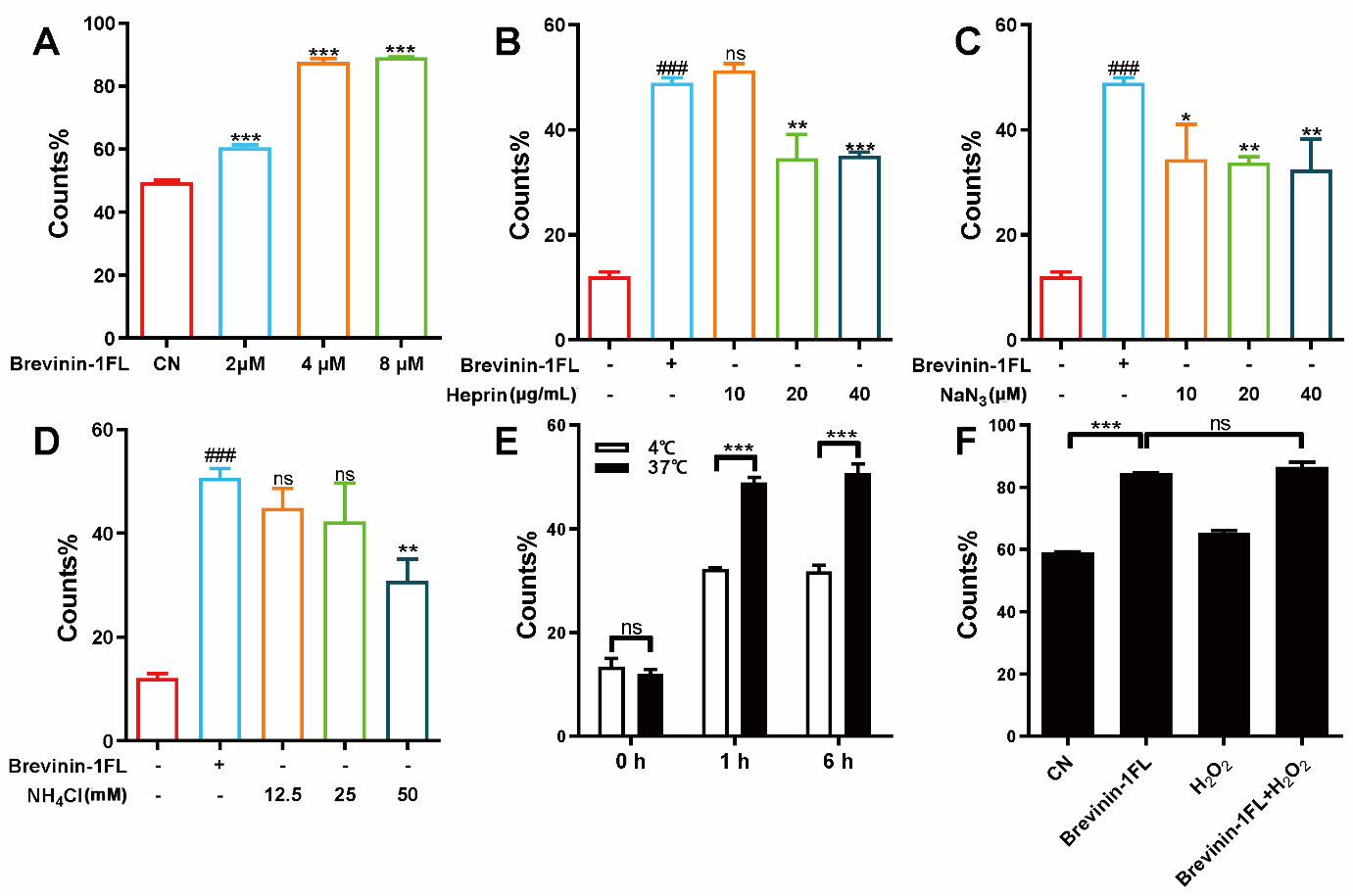
 FIGURE S2: Statistical analysis of Brevinin-1FL internalized into PC12 cells under different condition. Flow cytometry was carried out after PC12 cells were incubated with (A) FITC-labeled Brevinin-1FL, or (B-D) 8 μM FITC-labeled Brevinin-1FL plus heparin, NaN_3_, NH_4_Cl for 6 h, or (E) 8 μM FITC-labeled Brevinin-1FL at the indicated temperature and time conditions or (F) 8 μM FITC-labeled Brevinin-1FL in the presence or absence of 0.25 mM H_2_O_2_ for 6 h at 37˚C. CN: cells were treated with free FITC. Data presented are mean ± SD (n = 3). ^###^p < 0.001 compared to the control group; ns: no significant, ^*^p < 0.05, ^**^p < 0.01, ^***^p < 0.001 compared to the Brevinin-1FL group.

**
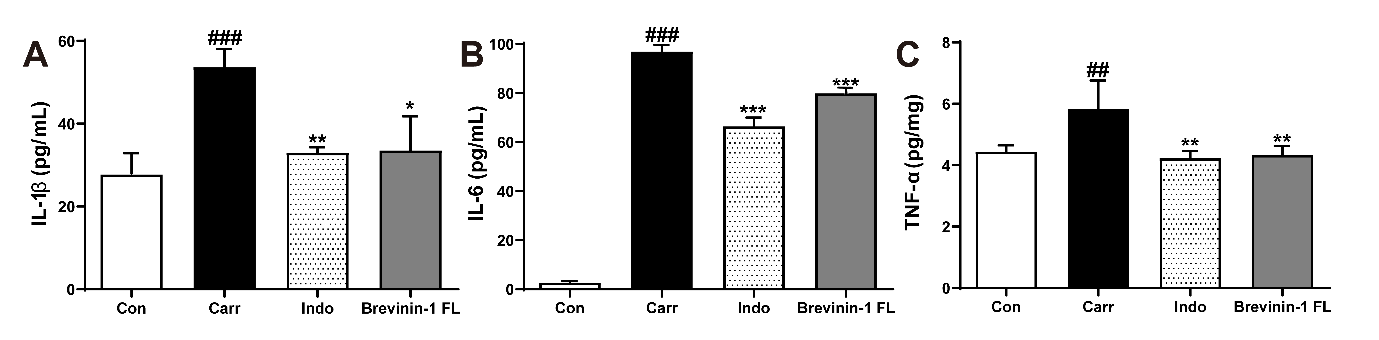
**FIGURE S3: Effects of Brevinin-1FL on the expression of IL-1β, TNF-α, and IL-6 in serum. Data presented are mean ± SEM (n = 3). ^##^p < 0.01, ^###^p < 0.001 compared to the control group; ^*^p < 0.05, ^**^p < 0.01, ^***^p < 0.001 compared to the model group.
